# Genome-Wide Analysis of Peptidoglycan Recognition Protein Genes in Fig Wasps (Hymenoptera, Chalcidoidea)

**DOI:** 10.3390/insects11090597

**Published:** 2020-09-04

**Authors:** Hong-Xia Hou, Meng-Yuan Guo, Jin Geng, Xian-Qin Wei, Da-Wei Huang, Jin-Hua Xiao

**Affiliations:** 1 Institute of Entomology, College of Life Sciences, Nankai University, Tianjin 300071, China; houhongxia003@126.com (H.-X.H.); gym9707@163.com (M.-Y.G.); gengjin122@163.com (J.G.); xianqin86@126.com (X.-Q.W.); 2 Key Laboratory of Zoological Systematics and Evolution, Institute of Zoology, Chinese Academy of Sciences, Beijing 100101, China

**Keywords:** PGRP, innate immunity, Toll, adaptive evolution, fig wasp

## Abstract

**Simple Summary:**

Insects live in a complex and diverse environment, threatened by a variety of microorganisms, and the innate immunity of which plays an important role in defending the invasion of pathogens. From an evolutionary perspective, different living environments and lifestyles drive the different evolutionary patterns of immune systems of insects. Fig wasps are closely associated with the fig syconia, divided into pollinators and non-pollinators according to whether they pollinate the figs. The pollinators are all herbivorous, and fulfil their development within the fig syconia, presenting different lifestyles and diets to non-pollinators, which lead to the chances of exposure to the pathogens varying greatly. The recognition of pathogens is the first step in innate immunity. Therefore, we focused on the different evolutionary patterns of peptidoglycan recognition protein genes between pollinators and non-pollinators, and found that the number of peptidoglycan recognition protein genes was significantly smaller than that of non-pollinators, and the initiation of Toll pathway of pollinators was simpler than that of non-pollinators. All the results suggested a streamlined innate immune recognition system of pollinators, and this information will provide more insights into the adaptive evolution of innate immunity in insects of host specificity.

**Abstract:**

The innate immunity is the most important defense against pathogen of insects, and the peptidoglycan recognition proteins (PGRPs) play an important role in the processes of immune recognition and initiation of Toll, IMD and other signal pathways. In fig wasps, pollinators and non-pollinators present different evolutionary histories and lifestyles, even though both are closely associated with fig syconia, which may indicate their different patterns in the evolution of PGRPs. By manual annotation, we got all the PGRP genes of 12 fig wasp species, containing seven pollinators and five non-pollinators, and investigated their putative different evolutionary patterns. We found that the number of PGRP genes in pollinators was significantly lower than in non-pollinators, and the number of catalytic PGRP presented a declining trend in pollinators. More importantly, PGRP-SA is associated with initiating the Toll pathway, as well as gram-negative bacteria-binding proteins (GNBPs), which were completely lost in pollinators, which led us to speculate that the initiation of Toll pathway was simpler in pollinators than in non-pollinators. We concluded that fig pollinators owned a more streamlined innate immune recognition system than non-pollinators. Our results provide molecular evidence for the adaptive evolution of innate immunity in insects of host specificity.

## 1. Introduction

The peptidoglycan recognition proteins (PGRP) are recognition proteins for bacterial peptidoglycan (PGN), playing important roles in the innate immunity of animals [1,2]. The first studied PGRP protein, found in the hemolymph of *Bombyx mori*, can specifically identify the peptidoglycan in the cell wall of gram-positive bacteria, and eventually activate the phenoloxidase, leading to melanization [3]. Thereafter, many PGRP gene families have been identified, e.g., 13 PGRPs in *Drosophila melanogaster* [4], 12 PGRPs in *B. mori* [5], seven PGRPs in *Anopheles gambiae* [6], and four PGRPs in *Apis mellifera* [7].

PGRPs contain at least one PGN-binding region, which is called the PGRP domain, with the length of approximately 165 amino acids, containing three α-helices and five β-folds, four of which are parallel, and the last one is antiparallel. The overall scaffolds of PGRPs are similar, the structures and activities of which were maintained by some conserved disulfides [8]. PGRPs are divided into two types, long-type PGRP (PGRP-L) and short-type PGRP (PGRP-S), according to the transcript lengths. The short-type PGRPs are generally distributed extracellularly, with signal peptides at the N-terminal, while the long-type PGRPs are widely distributed in the cell, the N-terminal of which contains transmembrane domains, and RIP homotypic interaction motif (RHIM) region, etc. According to the function, PGRPs can also be divided into catalytic PGRP and non-catalytic PGRP types. The catalytic PGRPs are conserved in their PGN-binding groove with a Zn^2+^-binding region, coordinated by three resides (corresponding to His_42_, His_152_, Cys_160_ in Dmel-PGRP-LB) [9]. The hydrolysis process of PGN by catalytic PGRP is generally believed to be that the Zn^2+^ binding in PGN-binding groove works as an electrophilic catalyst, which promotes the cleavage of the amide bond between MurNAc and L-Ala [10,11]. Some catalytic PGRPs have the ability to kill bacteria, for example, Dmel-PGRP-SB1 can kill 50% of *Bacillus megaterium* within ten minutes in the presence of Zn^2+^ [12]. In comparison, due to the lack of key resides binding to Zn^2+^, non-catalytic PGRPs only work as receptors in the immune response, by combining with PGN, but not hydrolyzing [11].

As described above, some PGRPs can specifically recognize the peptidoglycan of bacteria and initiate corresponding innate immune signal pathways. For example, in *D. melanogaster*, PGRP-SA and PGRP-SD, as well as the gram-negative bacteria-binding protein (GNBP), mainly recognize the peptidoglycan of most gram-positive bacteria (Lys-type PGN), activate serine protease, cleave the Spätzle precursor into mature Spätzle, and then activate the Toll pathway [13,14,15]. The transmembrane receptor protein PGRP-LC mainly recognizes the peptidoglycan of most gram-negative bacteria (DAP-type PGN) and activates the IMD pathway [16]. PGRP-LE is distributed both intracellularly and extracellularly in *D. melanogaster*. The extracellular PGRP-LE forms a complex with PGRP-LC to activate the IMD pathway, while the intracellular PGRP-LE interacts directly with Imd to activate the IMD pathway in a PGRP-LC-independent way [17,18]. As immune recognition proteins, PGRPs play key roles in the recognition of pathogens, the regulation of signal pathways and sterilization, which are of great significance to the study of an innate immunity of insects.

Fig wasps (Chalcidoidea, Hymenoptera) are the general name of all hymenopteran insects whose development and living are closely associated with the fig syconia—fruits of the fig trees (*Ficus*, Moraceae) [19]. According to whether they pollinate the figs, fig wasps are divided into pollinators and non-pollinators. The pollinators have undergone about 75 million years of coevolution with figs, and the association of the non-pollinators with figs may be due to independent colonization events [20]. The pollinators and non-pollinators present different lifestyles, even though both of them have close association with figs. The pollinators are all herbivorous, and they fulfil their development within the fig syconia by feeding on the endosperm of figs and mating inside the fig syconia, and then the females get out of the figs and directly enter another suitable fig to lay eggs and pollinate at the same time, but the males die after mating in their natal figs. While the diets of non-pollinators are more complex, including both herbivorous and carnivorous, upon maturation, most of them will get out of the fig syconia and finish the processes of mating and laying eggs outside of the figs.

The difference in lifestyles presented between pollinators and non-pollinators gives us a hint that, throughout their life history, the chances and duration of exposure to the open environment vary greatly between them. The pollinators may live in a more stable environment than the non-pollinators, and this may reflect their different innate immune recognition system. Based on the genome sequences of 12 fig wasp species, containing seven pollinators and five non-pollinators (Table S1), we acquired all of their PGRP genes using the method of manual annotation, and found that the number of PGRP genes of pollinators was significantly smaller than that of non-pollinators, and the initiation of the Toll pathway of pollinators was simpler than that of non-pollinators, which indicated that the putative different evolutionary patterns adapt to the different living environment.

## 2. Materials and Methods

### 2.1. The Genome Source of Fig Wasps

The genome sequences we used were submitted data from our lab (project accession PRJNA641212 and PRJNA494992).

### 2.2. The Identification of PGRP Gene Families of Fig Wasps

We got PGRP gene families of 12 fig wasps by manual annotation, based on the PGRP amino acid sequences of *D. melanogaster* [4], *A. mellifera* [7], and *Nasonia vitripennis* [21], downloaded from NCBI. Local *tblastn* was conducted to obtain the genome segment with the best match (*e* value ≤ 10^−5^) from the fig wasps. The complete gene structures and sequences were verified by Integrative Genomics Viewer (IGV). Softberry (http://www.softberry.com/berry.phtml?topic=fgenes_plus&group=programs&subgroup=gfs) was used to predict the gene structures for the PGRP genes, which lacked certain information from IGV.

### 2.3. The Characteristic Analysis of PGRP Genes

We identified PGRP domains by the NCBI-conserved domain database (https://www.ncbi.nlm.nih.gov/Structure/cdd/wrpsb.cgi), and presented some conserved sites using WebLogo 3 (http://weblogo.threeplusone.com/). The N-terminal signal peptides were predicted by SignelP (http://www.cbs.dtu.dk/services/SignalP/) and the transmembrane domain were predicted by TMHMM (http://www.cbs.dtu.dk/services/TMHMM/). Mapchart was used to describe the location of PGRPs on the genome, and MCScanX was used for collinearity analysis.

### 2.4. The Statistics and Phylogenetic Analysis of PGRP Gene Families

The R software was used to test whether the number of PGRP genes has a significant difference (*p* < 0.05) between the pollinators and non-pollinators. The MAFFT software was used for sequence alignment, and IQtree was used to predict the optimal model and construct a phylogenetic tree, and the bootstrap values were calculated with 1000 replicates. Finally, we used the Interactive Tree of Life (iTOL) (http://itol.embl.de/) to show the tree.

## 3. Results

### 3.1. Greatly Reduced Number of PGRP Genes in Pollinators than in Non-Pollinators

We obtained all of the PGRP gene members from the genomes of the 12 fig wasp species by manual annotation, and detected that the number of PGRP genes of pollinators was significantly smaller than that of non-pollinators (Wilcoxon test, *p* < 0.01) (Table 1). Specifically, for example, a pollinator species of the *Eupristina koningsbergeri* has only two PGRP genes, while the non-pollinator species of *Sycophila* sp.2 has 13. A gene tree was constructed using all the PGRPs from the insect species including *D. melanogaster* (Dmel-), *A. mellifera* (Amel-), *N. vitripennis* (Nvit-)*, Pteromalus puparum* (Pp) and the 12 fig wasps (Figure S1), which indicated the clustering pattern of these PGRP genes. By using this gene tree, we could identify the types of various PGRP genes and some specific lineages of the pollinators and non-pollinators through their gene clustering patterns. For example, we could clearly identify the catalytic PGRP genes (Clade I); although there was a specific lineage (Clade VI) in pollinators, there were more specific lineages (Clade II, IV, and V) in the non-pollinators.

### 3.2. The Location of PGRPs on the Genomes of the Fig Wasps Declaring Tandem Duplication Events

**Figure 1:**
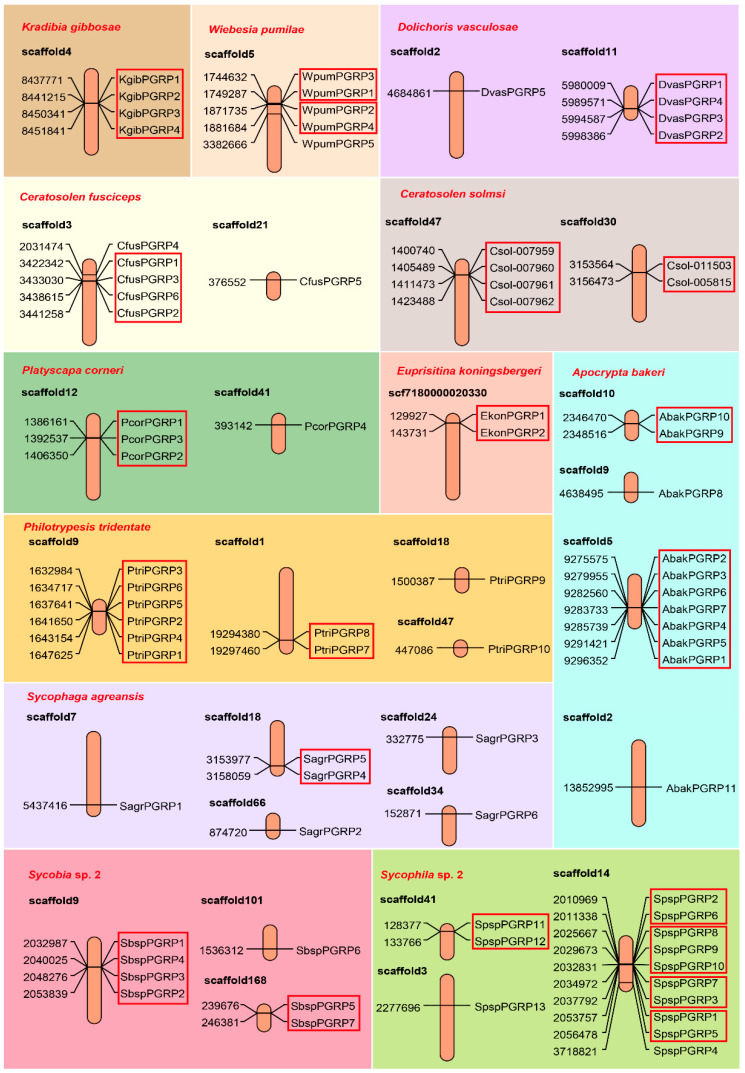
Schematic image showing the genomic localizations of the PGRP genes on the genomes of the 12 fig wasps. The location patterns could well declare tandem duplication events of PGRP genes in most fig wasp species. Each box of background color presented the results of one fig wasp species, and the orange columns are scaffolds. The gene names are listed in the right (the tandem genes were enclosed in red boxes), and their locations (the number represented the position of the gene start position on the scaffold) are marked on the left.

Gene family is a group of genes derived from a common ancestor, consisting of two or more copies from gene duplication or doubling. They show obvious similarities in structure and function, and code similar protein products [22,23]. For all of the PGRP genes from the fig wasps, we mapped their locations on their respective genomes and detected the events of tandem duplication (Figure 1). With the exception of the non-pollinator species of the *Sycophaga agraensis* (six PGRP genes sporadically distributed on five scaffolds), in most of the genomes of the fig wasps, the PGRPs were mainly concentrated on one scaffold, presenting a sequential distribution pattern. For example, in the species of *Sycophila* sp.2, there were, altogether, 10 out of 13 PGRP genes located on the scaffold of No.14, divided into four clusters, with each cluster containing at least two repetitive PGRP genes. In the species of *Kradibia gibbosae*, there were four PGRP genes, which were altogether located on the scaffold of No.4 as one cluster. We thus speculated that the PGRP gene families of wasps had expanded mainly through gene tandem duplication.

### 3.3. Significantly Streamlined Catalytic PGRPs in Pollinators Than in Non-Pollinators

**Figure 2:**
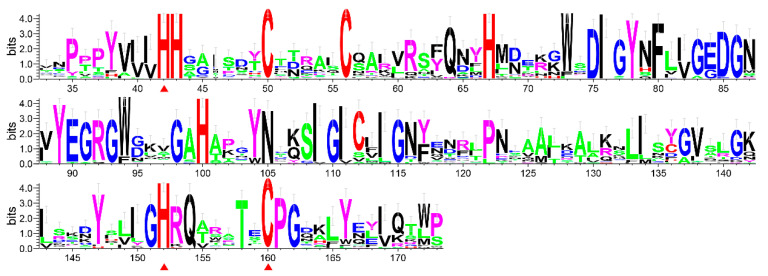
Conserved amino acid sites of the catalytic PGRP protein sequences from the insect species of *D. melanogaster*, *A. mellifera*, *N. vitripennis*, *P. puparum* and the 12 fig wasp species. The three key conserved Zn^2+^-coordinating residues of His_42_, His_152_ and Cys_160_ were marked with red triangles (Number of location sites were taken from the gene of Dmel-PGRP-LB from *D. melanogaster*).

**Figure 3:**
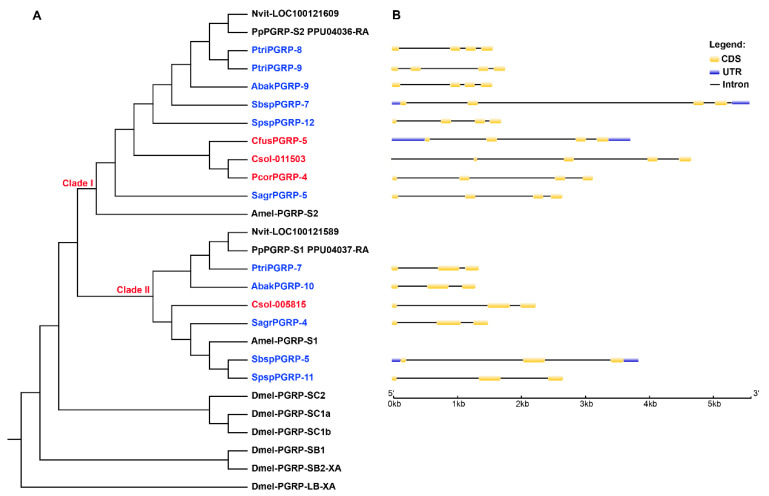
A gene tree of the catalytic PGRP genes (**A**) and their gene structures (**B**). (**A**) In the tree, the catalytic PGRPs of wasps were located on two clades of Clade I and Clade II. All of the five non-pollinator species had at least two catalytic PGRP gene members located on each clade, while only three of the studied seven pollinator species had catalytic PGRP genes. The red gene names represented pollinators, and blue represented non-pollinators. All of the catalytic PGRPs amino acid sequences from *D. melanogaster* (Dmel-), *A. mellifera* (Amel-), *N. vitripennis* (Nvit-), *P. puparum* (Pp) and the 12 fig wasps were used for Maximum Likelihood (ML) tree construction, with the model of WAG + I + G4. (**B**) Most of the catalytic PGRP genes in Clade I had four exons, while those in Clade II had three. Only catalytic PGRPs from the fig wasps were used to draw the gene structures.

PGRP genes are divided into catalytic PGRPs and non-catalytic PGRPs according to whether they have catalytic functions. In all of the PGRP sequences of the fig wasps, we screened out 15 catalytic PGRPs, all of which contained three Zn^2+^-coordinating residues in the PGN-binding groove (His, His, Cys) (Figure 2). We constructed a PGRP gene tree using all the catalytic PGRPs of the 16 species, in which the genes from wasps were located in two clades (Clade I and Clade II) (Figure 3A). The Clade I contained one PGRP gene from each of the species of *A. mellifera*, *N. vitripennis*, *P. puparum*, four non-pollinators (*S. agraensis*, *Sycobia* sp.2, *Sycophila* sp.2 and *Apocrypta bakeri*), and three pollinators (*Platyscapa corneri*, *Ceratosolen solmsi* and *Ceratosolen fusciceps*), and two PGRP genes from the non-pollinator species of *Philotrypesis tridentata*. Clade II contained one PGRP from each of the species of the *A. mellifera*, *N. vitripennis*, *P. puparum*, five non-pollinators, and one pollinator species of the *C. solmsi*. Overall, the number of catalytic PGRPs was significantly different between pollinators and non-pollinators (Wilcoxon test, *p* < 0.01) (Table 2), because each non-pollinator had at least two catalytic PGRPs (species of the *P. tridentata* even had three), located on each of both clades (Clade I and Clade II) in the gene tree, nevertheless four out of seven pollinators (*E. koningsbergeri*, *Wiebesia pumilae*, *Dolichoris vasculosae*, and *K. gibbosae*) had no catalytic PGRPs. Thus, compared to non-pollinators, the number of catalytic PGRPs in pollinators showed a decreasing trend. We further investigated the structure characteristics of the genes on both PGRPs clades, and found that genes in Clade I generally had at least four exons, while genes in Clade II had only three exons (Figure 3B).

### 3.4. Loss of PGRP-SA Genes and GNBP Genes Associated with the Initiation of Toll Pathway in Pollinators

In the gene tree of all PGRP genes from the studied species (Figure S1), we found that all the PGRPs clustered with Dmel-PGRP-SA and Amel-PGRP-S3 were from non-pollinators, which indicated that the pollinator species had lost the orthologous genes. It was known that Dmel-PGRP-SA and Amel-PGRP-S3 were associated with the initiation of the Toll pathway [7,13]. In addition, in another study, we found that the GNBPs (working together with PGRP-SA to initiate the Toll pathway) of pollinators were also completely lost, while the GNBPs of non-pollinators were present [24]. These findings caused us to speculate that the pollinator species had a reduction in initiation of the Toll pathway.

**Figure 4:**
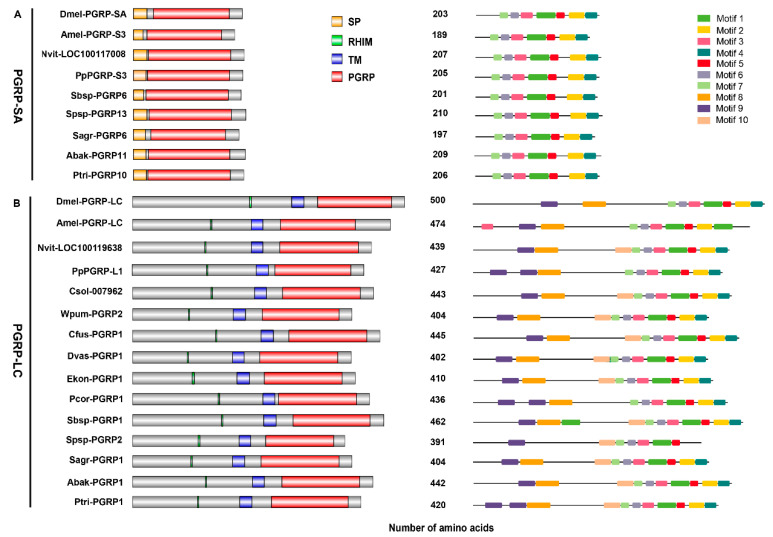
Domain and motif structure diagrams of PGRP-SAs (**A**) and PGRP-LCs (**B**). The domains were shown on the left (SP: signal peptide, RHIM: RIP homotypic interaction motif, TM: transmembrane domain, PGRP: peptidoglycan recognition protein), the motifs were marked with different colors on the right, and the number in the middle represented the length of amino acid of each PGRP. All of the PGRPs were from *D. melanogaster* (Dmel-), *A. mellifera* (Amel-), *N. vitripennis* (Nvit-), *P. puparum* (Pp) and the 12 fig wasp species.

**Figure 5:**
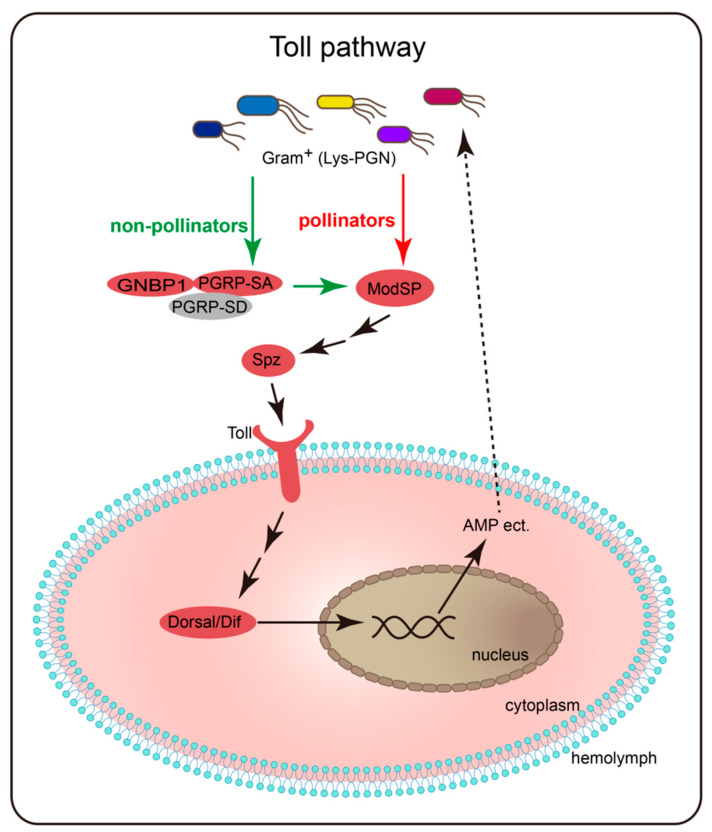
A schematic diagram of initiation of the Toll pathway among pollinators and non-pollinators. The putative recognition pathway of pollinators is indicated by red arrow, while that of non-pollinators is indicated by green arrows.

Meanwhile, we also looked into whether the PGRP-SA of non-pollinators had complete functional structures or not. By conducting motif and domain analysis on them, we found that they all contained the same and conserved motifs, complete N-terminal signal peptides and C-terminal PGRP domains (Figure 4A). Therefore, the non-pollinators might have similar patterns regarding the initiation of Toll pathway to other insects, while pollinators might have lost the conserved patterns (Figure 5).

### 3.5. PGRP-LC Genes Associated with the Initiation of IMD Pathway Harbored by All Fig Wasps

Due to the major differences in the initiation of Toll pathway between pollinators and non-pollinators, we also wondered if there were differences in the gene members in the initiation of IMD pathway between both fig wasp groups. When searching for the genes of PGRP-LC that play important roles in signal transduction of IMD pathway, we found that excepting the pollinator species of *K. gibbosae*, all the fig wasps had complete PGRP-LC genes (Figure S1), with encoded proteins including RHIM, the transmembrane domain (TM) and the PGRP domain (Figure 4B). The *K. gibbosae* was a unique species in the studied fig wasps, with a smaller genome (230.3 Mb), and most of the important gene members of IMD pathway, such as *Imd*, *FADD*, *Dredd*, *Tak1*, *Relish*, and *Pirk*, were absent [24]. Therefore, it is not unexpected to lose PGRP-LC in the *K. gibbosae*.

## 4. Discussion

In the innate immune system of insects, the recognition of pathogens is the first step, in which peptidoglycan recognition proteins play important roles [25]. The complexity of the insect immune system is closely related to its lifestyle and surrounding environment. The complex living environment drives the insects to evolve a strong immune system to resist the invasion of pathogens, while the superior living conditions greatly reduce insects’ chances of contact with pathogens, so their immune systems are relatively streamlined. For example, possessing a strong immune system in the American cockroach, *Periplaneta americana*, the GNBP and Toll families show great expansion, among which the number of GNBPs is the largest among all insects to date, and PGRPs (PGRP-LB, PGRP-LE) related to the IMD pathway are also expanded [26]. The diamondback moth, *Plutella xylostella* even has two PGRP-SAs in the same scaffold, serving a function in Toll pathway for the surveillance of Gram-positive bacteria, and possess a dramatic expansion of GNBPs, suggesting the function with diverse [27]. *B. mori* possesses some unique recognition genes and antimicrobial peptide genes, which do not exist in *Drosophila*, *Anopheles*, *Apis*, and *Tribolium* genomes, indicating that lepidopterans have a lineage-specific genetic evolution in immune recognition [5]. In addition, the number of immune genes from social insects *A. mellifera* is small, about a third of that of the fruit fly or *Anopheles*, which may be the strong social barrier preventing honey bees from being invaded by microorganisms, or the result of evolution with limited pathogens [7]. Compared to *Drosophila*, the decreasing diversities of PGRPs in hematophagous insects, such as the tsetse fly, reflect the relatively sterile environment during their development [28,29]. Furthermore, by feeding on nutrient-rich sap from the stems of plants, *Acyrthosiphon pisum* have lost all of their PGRPs [30]. The diverse of PGRPs may reflect the amount these insects are exposed to microbes [29]. In the present study of the pollinator and non-pollinating fig wasps, we found that the number of PGRPs, including the catalytic PGRPs, was significantly reduced in pollinators compared with non-pollinators. More importantly, we noticed that PGRP-SAs and GNBPs associated with the initiation of the Toll pathway were totally absent in the pollinators, but still retained in the non-pollinators. Nothing like this had ever been found before. Although the PGRPs are absent in the daphnia, *Daphnia pulex*, the expansion of another recognition protein GNBP may compensate for the absence of PGRPs [31]. We thus speculated that the immune recognition modes of pollinators tended to be extremely streamlined due to their long-term co-evolutionary history with figs.

It is interesting that the *K. gibbosae* had relatively few PGRP gene members (four genes) in the studied fig wasp species, and it had lost many crucial genes of IMD pathway [24]. Some specialized insects also present such kind of pattern. For example, the aphid, *A. pisum*, has lost PGRP genes and IMD pathway, but presents other intact immune pathways (Toll, JNK, JAK/STAT) [30]. The body louse, *Pediculus humanus*, who lives on human blood, possesses only one PGRP gene, has lost gene members in IMD pathway, but retains other intact immune pathways [32]. Besides, it is common that multiple standard components of the IMD pathway are absent in Hemiptera [33]. Many hemipteran species feed on phloem sap of plants or blood, reducing the possibility of pathogen intake and thus eliminating the necessity for specific immune defenses in the gut. What these situations have in common are fewer pathogens in their food (free-of-microbes diets) [33]. In short, the host specificity may be related to the streamlined PGRP genes and IMD pathway, but why other pollinator species that also present host specificity have not lost so many gene members of PGRP and IMD pathway still confuses us. Maybe all the pollinators are showing the pattern of losing of these gene members, but the loss in the species of the *K. gibbosae* is more obvious. Further comparative analysis of more species will help us unravel the mystery.

## 5. Conclusions

Fig wasps are a special group of insects protected by fig syconia. Even though both pollinators and non-pollinating fig wasps are closely associated with figs, their different evolutionary histories and lifestyles may indicate different patterns in immune recognition, such as the evolution of peptidoglycan recognition proteins (PGRPs). We found that the PGRP gene family of fig wasps was mainly expanded by tandem duplication, however the number of PGRP genes of pollinators was significantly smaller than that of non-pollinators, and the catalytic PGRPs were gradually lost in pollinators. In addition, PGRP-SAs associated with the initiation of Toll pathway were all lost in pollinators, but still retained in the non-pollinators. Based on these results, we speculated that the innate immune recognition systems of pollinators were more streamlined than non-pollinators. These differences might be attributed to their longer history of adaptive evolution to the living environment within fig syconia.

## Figures and Tables

**Table 1:** The number of peptidoglycan recognition protein (PGRP) genes of pollinators and non-pollinators.

Group	Name	Gene Number	Significance
Pollinators	*Eupristina koningsbergeri*	2	Wilcoxon test, ** p = 0.0086, *p* < 0.01
	*Kradibia gibbosae*	4	
	*Platyscapa corneri*	4	
	*Wiebesia pumilae*	5	
	*Dolichoris vasculosae*	5	
	*Ceratosolen solmsi*	6	
	*Ceratosolen fusciceps*	6	
Non-pollinators	*Sycophaga agra* *ensis*	6	
	*Sycobia* sp.2	7	
	*Philotrypesis tridentata*	10	
	*Apocrypta bakeri*	11	
	*Sycophila* sp.2	13	

Note: ** represents extremely significant difference between the gene numbers of the pollinator and non-pollinator groups (*p* < 0.01).

**Table 2:** The number of catalytic PGRP genes of pollinator and non-pollinator species.

Group	Name	Gene Number	Significance
Pollinators	*Eupristina koningsbergeri*	0	Wilcoxon test, ** p = 0.0099, *p* < 0.01
	*Kradibia gibbosae*	0	
	*Platyscapa corneri*	1	
	*Wiebesia pumilae*	0	
	*Dolichoris vasculosae*	0	
	*Ceratosolen solmsi*	2	
	*Ceratosolen fusciceps*	1	
Non-pollinators	*Sycophaga agra* *ensis*	2	
	*Sycobia* sp.2	2	
	*Philotrypesis tridentata*	3	
	*Apocrypta bakeri*	2	
	*Sycophila* sp.2	2	

Note: ** represents extremely significant difference between the gene numbers of the pollinator and non-pollinator groups (*p* < 0.01).

## Supplementary Materials

The following are available online at https://www.mdpi.com/2075-4450/11/9/597/s1, Figure S1: A gene tree of the PGRP genes. Table S1: The fig wasp species of pollinators and non-pollinators used in this study.
