# Supplementary material for: Genome-Wide Analysis of Peptidoglycan Recognition Protein Genes in Fig Wasps (Hymenoptera, Chalcidoidea)
Source: Insects. 2020 Sep 4;11(9):597. doi: 10.3390/insects11090597 (PMC7565001; doi:10.3390/insects11090597)
Supplement: Supplementary file 1 [file insects-11-00597-s001.pdf]

Genome-wide Analysis of Peptidoglycan Recognition Protein Genes in Fig Wasps (Hymenoptera, Chalcidoidea)

Hong-Xia Hou [1], Meng-Yuan Guo [1], Jin Geng [1], Xian-Qin Wei [1], Da-Wei Huang [1,2]*, Jin-Hua Xiao [1]*

Supplementary Materials:

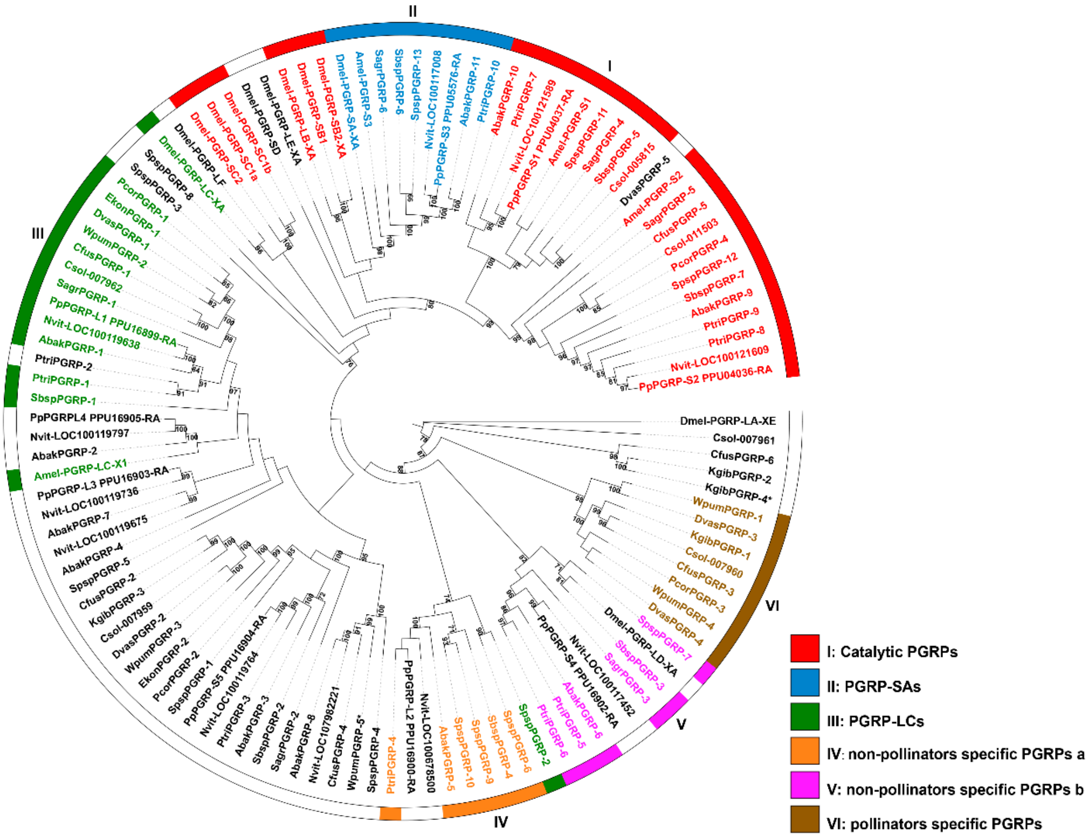

Figure S1. A gene tree of the PGRP genes. The PGRPs clustered with Dmel-PGRP-SA and Amel-PGRP-S3 were only from non-pollinators. All of the amino acid sequences of the PGRPs from species of *Drosophila melanogaster* (Dmel-), *Apis mellifera* (Amel-), *Nasonia vitripennis* (Nvit-), *Pteromalus puparum* (Pp) and 12 fig wasps were used for Maximum Likelihood (ML) tree construction, with the model of VT+R6. The red indicated PGRPs with catalytic activity, the blue indicated PGRPs associated with the initiation of Toll pathway, the green indicated PGRPs associated with the initiation of IMD pathway, the orange and purple indicated PGRPs with non-pollinator-specificity, and the brown indicated PGRPs with pollinator-specificity. * represented the PGRP genes with an uncomplete domain in the C terminal or N terminal.

Table S1. The fig wasp species of pollinators and non-pollinators used in this study.

**Group**	**Name**	**Abbreviation**	**Diet**	**Oviposition location**
Pollinators	*Ceratosolen solmsi*	Csol	phytophagous	inside figs
	Kradibia gibbosae	Kgib	phytophagous	inside figs
	Wiebesia pumilae	Wpum	phytophagous	inside figs
	Ceratosolen fusciceps	Cfus	phytophagous	inside figs
	Dolichoris vasculosae	Dvas	phytophagous	inside figs
	Eupristina koningsbergeri	Ekon	phytophagous	inside figs
	Platyscapa corneri	Pcor	phytophagous	inside figs
Non-pollinators	*Sycobia* sp.2	Sbsp	phytophagous	outside figs
	Sycophila sp.2	Spsp	parasitic or phytophagous	outside figs
	Sycophaga agraensis	Sagr	phytophagous	outside figs
	Apocrypta bakeri	Abak	parasitic or phytophagous	outside figs
	Philotrypesis tridentata	Ptri	parasitic or phytophagous	outside figs
